# Fertility Preservation for Cancer Patients: A Review

**DOI:** 10.1155/2010/160386

**Published:** 2010-03-31

**Authors:** Tosin Ajala, Junaid Rafi, Peter Larsen-Disney, Richard Howell

**Affiliations:** ^1^Department of Obstetrics and Gynaecology, Basingstoke and North Hampshire NHS Trust, Basingstoke RG24 9NA, UK; ^2^Department of Obstetrics and Gynaecology, Brighton and Sussex University Hospitals, Eastern Road, Brighton BN2 5BE, UK

## Abstract

Infertility can arise as a consequence of treatment of oncological conditions. The parallel and continued improvement in both the management of oncology and fertility cases in recent times has brought to the fore-front the potential for fertility preservation in patients being treated for cancer. Oncologists must be aware of situations where their treatment will affect fertility in patients who are being treated for cancer and they must also be aware of the pathways available for procedures such as cryopreservation of gametes and/or embryos. Improved cancer care associated with increased cure rates and long term survival, coupled with advances in fertility treatment means that it is now imperative that fertility preservation is considered as part of the care offered to these patients. This can only be approached within a multidisciplinary setting. There are obvious challenges that still remain to be resolved, especially in the area of fertility preservation in prepubertal patients. These include ethical issues, such as valid consent and research in the area of tissue retrieval, cryopreservation, and transplantation.

## 1. Introduction

Infertility can arise as a consequence of treatment of oncological conditions. The parallel and continued improvement in both the management of oncology and fertility cases in recent times has brought to the fore-front the potential for fertility preservation in patients being treated for cancer. 

Infertility can arise as a consequence of treatment of oncological conditions. The parallel and continued improvement in both the management of oncology and fertility cases in recent times has brought to the fore-front the potential for fertility preservation in patients being treated for cancer. With the publication of NICE Guidance on the applications of cryopreservation in cancer treatment [1], the emphasis on life style versus health issues within fertility care for cancer patients must shift. Clearly Oncologists must be aware of situations where their treatment will affect fertility in patients who are being treated for cancer and they must also be aware of the pathways available for procedures such as cryopreservation of gametes and/or embryos. The NICE Guidance has developed on the back of a working party from the Royal College of Physicians and the Royal College of Radiologists, which have recommended the procedures that are to be followed before commencing chemotherapy and radiotherapy which are likely to affect fertility; and also the management of post treatment infertility. The British Fertility Society has produced a strategy for developing policy and practice in fertility preservation for survivors of cancer [2]. 

It must be remembered that the NHS will currently only fund one cycle of IVF for any woman with infertility and this is only available to women who satisfy strict inclusion criteria usually including no previous children to either partner, age between 23 to 39 years and a body mass index less than 30.

## 2. The Impact of Oncology Therapy on Fertility

### 2.1. Surgical Management

Surgery can impact on fertility in one of two ways. It can either render someone infertile by removal of reproductive organs or in the case of the male it can interfere with potency or ejaculation. There is no doubt, however, that in recent years there has been a tendency towards more conservative treatment for many malignancies affecting the reproductive organs.

#### 2.1.1. Female Patients

In women, there has been tendency towards less radical approaches to cancer of the cervix with the development of loop excision techniques for very early cervix cancer and more recently the development of the radical trachelectomy [3, 4] which allows a radical approach to cervix cancer that is treatable surgically, but with preservation of the uterus and thus fertility. Endometrial cancer is usually a disease of the postmenopausal group or at least in those who have had completed their family, and therefore it is unusual for treatment of this disease, which does involve hysterectomy and bilateral oophorectomy, to impact upon fertility. Epithelial ovarian cancer continues to be treated radically with loss of reproductive organs but increasing understanding of germ cell malignancies and borderline tumours of the ovary has led to a more conservative approach to these neoplasms and often a single oophorectomy will be performed where in the past, a hysterectomy and/or bilateral oophorectomy would have been the treatment of choice. It is unusual for vulval carcinoma to be seen in the reproductive age group, and although it may have major psychosexual impact it would be unusual for a surgical approach to these to impact upon fertility.

#### 2.1.2. Male Patients

Testicular cancer is the most prevalent cancer affecting the reproductive organs of the male, and tends to occur at an age where child bearing remains a potential issue. The majority of these are treated with a unilateral orchidectomy [5, 6] and staging thus preserving reproductive capacity. Other tumours affecting the reproductive organs in the male tend to occur in a much older age group where fertility issues may not be of importance. Surgery for other pelvic malignancy such as bladder, prostate, and rectum can clearly interfere with potency or ejaculation, however, the age dependency of these tumours would suggest that most occur in an older group of patients where fertility issues are unlikely to be of major importance. Thus fertility services are unlikely to be as important to this older group of individuals.

## 3. Chemotherapy Effects on Fertility

Chemotherapy can produce significant effects upon patient fertility. These effects are dependent on a number of factors [7]:

- *radical versus adjuvant chemotherapy*. Radical chemotherapy generally has more profound effects on fertility than adjuvant chemotherapy,
- *single agent versus combination chemotherapy*. Increasing complexities of regimes are more likely to have impacts upon fertility than single agent,
- *dose-dependent effects*. Increasing doses are likely to have more profound effects on fertility than lower doses,
- *drug-dependent effects*. Different agents have a markedly different impact upon fertility with some chemo-therapeutic agents sparing fertility whilst others are extremely toxic in this regard,
- *age-dependent effects*. In the female in particular, age has a profound effect on chemotherapy toxicity. Women administered chemotherapy under the age of 40 have a much higher chance of regaining the normal ovarian function whilst the majority of women over 40 administered toxic chemotherapy will be rendered menopausal by their treatment. Presumably part of the reason for this is the fact that the natural attrition rate of oocyte sees a large drop in oocyte numbers over age 40 (decreased ovarian reserve) and this corresponds with decreased live birth rates in fertility patients over the age of 40,
- *male versus female physiology*. The testis in the male is exquisitely sensitive to chemotherapy whereas, as has already been stated, the female is variable in terms of the tolerance to chemotherapy agents.

Detailed information regarding fertility effects of many chemotherapy regimes is lacking, but specific examples where chemotherapy affects fertility are documented include the following. 

### 3.1. Toxic Effects of Commonly Used Chemotherapeutic Agents on the Testis

Gonadal toxicity of the testis affects spermatogenesis more than it does testosterone production. This stems from the increased cyto-sensitivity of the germinal epithelium in comparism to that of the leydig cells. The germinal cell division is extremely high through increased meiotic and mitotic activity thus allowing for increase sensitivity to cytotoxic agents [8–10]. Sexual maturation of the testis also influences the degree of gonadal damage experienced when exposed to cytotoxic drugs, the prepubertal testis being less susceptible than post-pubertal testis [9]. The extent to which spermatogenesis is affected is influenced by the type of cytotoxic agent(s) and the dose to which it is exposed [8–10]. Table 1 illustrates the degree of gonadal dysfunction of commonly used cytotoxic agents.

### 3.2. Toxic Effects of Commonly Used Chemotherapeutic Agents on the Ovary

A fixed number of primordial follicles present at birth form the ovarian reserve into puberty. Postpuberty these primordial follicles contain single oocytes arrested in the prophase of the first meiotic division and are highly sensitive to cytotoxic drugs leading to cellular death [11]. Follicular depletion has been shown to be physiologically age dependent, the maximum rate of depletion occurring around the age of 38 years when the reserve is just about 10% the number present at menarche [12]. The gonadal toxic effect is thus not just dependent on type(s) and dosage of the cytotoxic drug(s) employed but also on the age of the woman. Cell cycle nonspecific agents such as cyclophosphamide (alkylating agent) will destroy resting primordial cells as opposed to cell cycle specific agents such as methotrexate (antimetabolite) which spare the rest primordial cells and as such are less gonadotoxic.

- *Adriamycin and cyclophosphamide* have a 38% ovarian failure rate in women aged over 40 years at 2 years post chemotherapy.
- *Cyclophosphamide, Hydroxlydaunorubicin (Adriamycin) Oncovin (vincristine), and Prednisolone* do not usually lead to permanent amenorrhoea in women under 40 years of age, but may lead to early menopause in older women.
- *ABVD (Doxorubicin, Bleomycin, Vincristine, and Dacarbazine)* used in the treatment of Hodgkin's disease is significantly less toxic in terms of fertility than the older *MOPP (Mechlorethamine, Vincristine, Procarbazine and Prednisolone). *
- *Bleomycin and doxorubicin* have minimal effects on fertility.
- *Vinca alkaloids and antimetabolites* have very mild effects on fertility (*Methotrexate* very mild at 6 gm total dose).
- *Taxanes* are not clearly defined in terms of their impact on fertility.
- *Cyclophosphamide, methotrexate, and 5-fluorouracil (CMF)* a classical breast cancer regime will render 71% of women over 40 years of age amenorrhoeic at 2 years.

## 4. Radiotherapy Effects on Fertility

The principle of radiotherapy is based on the ionisation of cellular atoms and molecules leading to the destruction of double and single DNA structures within the cell structure. A chain of events is set up, disrupting the cell-cycle leading to apoptosis of the cells. Radiotherapy has its use in oncology because unlike malignant cells, most normal cells have the inert ability to recover from the effects of radiotherapy. 

Clearly radiotherapy can be administered as external beam therapy (teletherapy), or as intracavity (brachytherapy) treatments. In addition to this, radiotherapy can be given with radical curative intent or as adjuvant therapy often postoperatively.

The direct effects of radiotherapy are dose dependent and are also dependent on the field applied to the individual. It is important to consider the effect of scattered radiation as well as direct irradiation when assessing likely effects on fertility. For example, although pelvic irradiation may not directly hit the testis in the male patient, scatter of radiotherapy will occur from this area which may have an impact on fertility [13, 14].

### 4.1. Female Patients

Radiotherapy effects on the female are dose dependant. The application of 14.3 Gray to an ovary in a woman over 30 years of age will usually render her irreversibly infertile and menopausal [15]. A dose of 6 Gray to the ovary of a woman less than 30 years of age is usually reversible, but ultimately, will bring the menopause forwards. Thus the female is not only concerned with issues regarding fertility but also with hormone production, as both seem to be equally affected by radiotherapy. 

Although the uterus is relatively resistant to radiotherapy there is no doubt that uterine irradiation is harmful [16] and even if fertility is conserved, uterine irradiation will result in poor implantation [17]. This appears to be due to a number of factors including reduced uterine volume and blood flow which have been demonstrated to result in increased mid-trimester losses, preterm labour and intrauterine growth retardation [18]. The vagina is relatively radio-resistant however, irradiation of this organ carries with it the risk of loss of lubrication and stenosis which may result in physical impairments to fertility as well as major psychosexual issues [19].

### 4.2. Male Patients

The effect of radiotherapy on male fertility is also dose dependent. The application of greater than 6 Gray to testes will result in irreversible azoospermia. At levels of 3.5 Gray, sterility does occur, but this is reversible although commonly such recovery will take 18 to 24 months. Treatment age (the younger the better) and normal pre-treatment sperm count influence the recovery rate [20]. Fractional radiotherapy to testes for treatment of carcinoma-insitu of the testis usually involves high doses of radiotherapy which lead to permanent azoospermia [21–23]. Interestingly, the Leydig cells of the testis seem far more resistant to radiation effect and therefore testosterone production is usually less impaired in patients receiving even relatively high dosages of radiotherapy relative to its effects on sperm production [24]. In addition, libido and erection will usually remain normal in the male and its sterility that is the main concern. However it is not unusual for patients who have had pelvic irradiation to suffer from erectile dysfunction as a long-term complication. This may in part be explained by radiation induced vascular disease leading to reduced blood flow in the pelvic and penile vessels [25].

## 5. Fertility Preservation for Female Cancer Patients

### 5.1. Options for Fertility Conservation

#### 5.1.1. Hormone Protection by Suppressing Ovaries

GNRH analogues have been utilised during chemotherapy to suppress ovarian cycling and induce a temporary medical menopause. The action of GnRH analogues is not clearly understood as primordial and primary follicles do not have GnRH receptors and it is possible that GnRH analogues preserve those follicles that have already initiated growth. Several studies in animals have suggested that protection may be offered by undertaking this manoeuvre [26], however its benefit in human therapy remains debatable. The human studies presently available are limited in design and lack of long-term follow-up [27]. The Option Trial (Ovarian Protection Trial in Oestrogen Non-Responsive Premenopausal Breast Cancer Patients Receiving Adjuvant or Neo-Adjuvant Chemotherapy) for breast cancer looked at hormone suppression using GNRH analogues and progestogens and examined the impact of this on fertility. It concluded that using goserelin concurrently with chemotherapy is associated with a high rate of ovarian function preservation [28].

#### 5.1.2. Ovarian Transposition (Oophorepexy)

The aim is to surgically remove the ovaries from the direct field of radiation. It is found useful during the treatment of gynaecological cases and haematogical cancers such as Hodgkin's lymphomas. Most ovarian transpositions are carried out laparoscopically and there have been suggestions that lateral transposition may be more protective than median transposition of the ovaries [29, 30].

#### 5.1.3. Storage by Cryopreservation of Embryos, Oocytes, or Ovarian Tissue

The choice of what tissue type should be preserved depends on the type of cancer, the patient's age, and whether she has a partner. Often it is time however that is the limiting factor in this choice.

### 5.2. Embryo Storage

Embryo storage is ideal for an adult woman in a stable relationship as it is an established technique which has been available since the mid 1980s. IVF offers a success rate of approximately 30% per cycle (dependent on age) and this is similar to the natural conception rate that is achievable by healthy couples without assisted reproductive techniques [31, 32]. It involves stimulating the ovaries using gonadotrophins which results in high oestrogen levels, and certainly this raises concerns for some tumours such as breast cancers with oestrogen receptor positivity. It is still unclear what the risks of such techniques in terms of tumour progression or relapse in a hormone dependent cancer are. Some groups have attempted to address this by using tamoxifen or letrozole alone or in combination with standard IVF stimulation for women with breast cancer [33] or endometrial cancer. Patient numbers are small and long-term studies are currently not available.

IVF stimulation takes a minimum of two to three weeks depending on a patient's menstrual cycle and could be anything up to five weeks. After stimulation of follicles to maturation an egg collection procedure is undertaken usually as a day case under sedation or a general anaesthetic where vaginal ultrasound probe is used to guide transvaginal collection of eggs. IVF is then undertaken to fertilise the patient's eggs with the partner's sperm before freezing the embryo. At present there is limited availability donor sperm for adult women trying to preserve reproductive potential whilst undertaking chemotherapy.

### 5.3. Oocyte Storage

This technique is suitable for adults and for older teenagers who do not have a current partner. It is important to realise that this is a new technique and success rates are low at present with perhaps less than 5% success rates achievable per cycle [34, 35]. Clearly this figure may rise with improved techniques in the future but at present less than 100 pregnancies have been documented worldwide using this technique. The technique involves stimulation of the ovaries, harvesting of eggs and then freezing them, which is technically very difficult. Stored eggs can later be thawed and IVF techniques with ICSI can be used. Oocytes are much more sensitive to damage from cryopreservation techniques than embryos (probably secondary to spindle damage from ice crystal formation). The formation of ice crystal and the attendant cellular damage during freezing can potentially be avoided by vitrification. For younger patients oocyte storage may be an option, as harvest techniques can include transabdominal ultrasound and laparoscopy for retrieval of eggs rather than subjecting the patient to the transvaginal technique.

### 5.4. Ovarian Tissue Storage

This is a technique that can be used for adults and for children but it is very much experimental at the present time. Optimal treatment benefit can only be expected in the presence of a healthy ovarian reserve, as such it less likely to be beneficial to the older patient above 40 years. Laparoscopy is required to undertake a biopsy of an ovary or to remove the whole ovary for preservation. The first case of an ovarian transplant operation was reported in 2000 [36]. (all cases of successful postimplantation pregnancy reported to date have utilised the whole cortical ovarian tissue) [37]. It is therefore an invasive procedure under general anaesthetic and carries a mortality rate of 1 in 12,000. Tissue obtained is cut into thin sections and then cryopreserved in a relatively straightforward fashion. Fewer than 15 patients world-wide have had their thawed ovarian tissue reimplanted via either orthotopic or heterotopic transplantation. Until recently there had been no case reported of a successful live birth after orthotopic transplantation of cryopreserved ovarian tissue. Donnez et al. [38] reported in The Lancet in 2004 a live birth after transplanting cryopreserved ovarian tissue back into the pelvis of a woman following treatment for Stage 4 non-Hodgkin's lymphoma and Meirow et al. in 2005 [39] reported a further live birth in another young woman who had also been treated for non-Hodgkin's lymphoma. In another case reported in 2005, laparoscopic cortical ovarian transplantation was carried out between a set of 24-year-old monozygotic twins. The recipient twin had documented clinical premature ovarian failure. Following ovarian transplantation, normal ovarian function was restored leading to conception and delivery of a live infant at 38 weeks [40].

The risk of re-implanting tissue with occult cancer while small remains significant. Only patients with cancer cases associated with low risk of ovarian metastasis such as squamous cell carcinoma of the cervix, Wilm's tumour, Hodgkin and non-Hodgkin's lymphoma should be considered for future autotransplantation. Patients with moderate and in particular high risk of ovarian involvement should not be considered for future autotransplantation [41].

## 6. Fertility Preservation in the Male Cancer Patient

Sperm cryopreservation remains the obvious choice for males capable of producing a semen sample. This is mainly achieved through masturbation, but can also be achieved through testicular biopsy and testicular sperm extraction (TESE) and epididymal aspiration of sperms to be used for ICSI in cases of azoospermia or ductal blockage. Sperm collection should be carried out prior to treatment to avoid collection of potentially abnormal DNA containing cells.

Other forms of potential male fertility preserving methods such as hormonal gonadoprotection and testicular tissue cryopreservation with subsequent transplantation have either been found unhelpful or still in the experimental stages [42]. Gonadal shielding is also limited in its value.

### 6.1. Practical Laboratory Issues for Sperm Banking

A critical factor for male patients requiring sperm banking is the timing of the sample as it is essential that this occurs before chemo- or radiotherapy is undertaken. Prescreening is required and the patients are checked for Hepatitis B and C, Syphilis, HIV and CMV. (Although positive results will not preclude a patient from undertaking sperm storage, a positive result will determine the batching and isolation required for semen storage.) Appropriate paperwork is then undertaken as required by the HFEA including the signing of the [HFEA (006)] MS consent form to storage of sperm.

Patients are then required to attend a Reproductive Medicine Centre to produce a semen sample (ideally this should be three samples a few days apart). Samples are then stored in liquid nitrogen at minus 196 degrees centigrade in two separate locations for 10 years. Each sample undergoes a standard diagnostic semen analysis and is assessed against standard criteria (WHO 2000).

### 6.2. Long-Term Considerations

Approximately 50% of sperm stored will be lost during the preservation and storage process. Potential damage to cryopreserved sperm includes osmotic injuries from cryoprotective agents, hypothermic injury [43] and oxidative damage [44].

Most patients receive an annual letter from the Andrology Unit storing their semen to check that the patient wants the sample to be retained in storage. For patients to be eligible for semen storage they must be less than 55 years of age, they must be able to give informed consent for storage, screening and the fate of the sperm. It is certainly feasible to store sperm for many years and, at present, patients are not charged for this facility but the issue of whether the NHS should be formally funding this remains unanswered.

## 7. The Challenge for Children

Examination of the trends in Five-Year-Survival Rates for the commonest childhood malignancies reveals sustained improvements in cure rates. Over the twenty-five year-period from 1964, the five year survival for acute lymphocytic lymphoma has risen from close to 0% to close to 70%, for non-Hodgkins lymphoma has risen from approximately 20% to almost 80% and for Wilms-tumour has risen from 25% to about 80% [45]. Even since that time, five-year survival rates have continued to rise slowly for these common forms of childhood malignancy. Although the challenge for poor prognosis tumours such as neuroblastoma remains, for other tumours the aim of the future is to sustain improvements in cure rates and to minimise the late effects for curable tumours. Preservation of fertility remains of great importance in these young patients and awareness of services available to them may reduce the long-term morbidity of their cancer treatment.

### 7.1. Young Male Patients

#### 7.1.1. Background

The testicle is responsible for spermatogenesis (the production of mature sperm). In addition to this, it carries out steroidogenesis (the production of steroid hormones including testosterone). Damage to the Leydig cells of the testis results in reduced testosterone production and an elevated luteinizing hormone levels (from the pituitary gland). Damage to the germinal epithelial of the testes results in elevated follicular stimulating hormone (FSH) levels, low inhibin B levels and impaired spermatogenesis. 

As previously discussed, a radiation dose of greater than 6 Gray to the germinal epithelium will result in permanent azoospermia. In the prepubertal male, irradiation greater than 20 Gray to the Leydig cells of the testis will cause significant damage in terms of testosterone production but in the post-pubertal male a level of greater than 30 Gray is required to cause this level of damage. 

A study by Thomson et al. [46] examined male fertility after childhood cancer (treated with radiotherapy or chemotherapy) and examined semen analyses in long-term cancer survivors (average of five years post treatment) compared with controls. In the control group (untreated), 85% of subjects had a normal semen analysis, approximately 10% had poor motility and 5% had oligospermia. The findings were quite different in the cancer survivors group: 30% of patients had normal semen analysis, almost 30% had poor motility, 15% had oligospermia and over 20% had azoospermia. Of the subjects who actually produced spermatozoa, it was clear that the concentration of sperm produced was significantly less than that of the control group. An analysis of sperm DNA integrity as a measure of quality of sperm showed no significant difference between the control and the cancer survivor groups. Thus, it can be concluded that subfertility associated with previous treatment of childhood cancer may be due to azoospermia or oligospermia where there is a significant reduction in sperm concentration but normal sperm quality.

#### 7.1.2. Strategies for Fertility Preservation in Young Males Undergoing Treatment for Cancer

Sperm banking remains the obvious choice for males capable of producing a semen sample, however young males will only start producing sperm cells suitable for cryopreservation around the age of 12-13 years [47]. Certainly sperm retrieval should be offered to patients in whom the risk of infertility is high, but there is now a good evidence base to suggest that if the testicular volume is less than 10 mls, it is very unlikely that the patient will demonstrate any significant spermatogenesis. Thus sperm retrieval should be limited to males where testicular volume is greater than 10 mls and samples should ideally be produced by ejaculation. In the situation where young males are unable to ejaculate then rectal electro stimulation [48] or testicular/epididymal [49] aspiration may be offered and can be successfully undertaken. Sperm banking can then be done with the expectation that the semen can be used at a later date. At present, the later use of stored sperm is likely to require assisted conception methods such as intracytoplasmic sperm injection (ICSI) to optimise the likelihood of successful fertilisation [49].

#### 7.1.3. Questions Related to Sperm Storage

A number of questions are raised by the opportunity to provide sperm storage for young male cancer patients.

1. Who exactly will need it?
2. Who will raise the issue of sperm storage with the patient?
3. Where will the patient produce the sample for storage?
4. When, in relation to his treatment, should the patient produce a sample? Situations arise where a patient is too ill or indeed has to be treated so acutely that there is insufficient time to offer this option?
5. Is it appropriate to discuss the issue of sperm storage with a patient who is struggling to cope with his diagnosis and forthcoming treatment?
6. What is the cost of storage of sperm?

At present there is a one year audit being undertaken of all 22 United Kingdom Children's Cancer Study Group (UK CCSG) centres which is Multi-Centre Research Ethics Committee (MREC) and The Human Fertilisation and Embryology Authority (HFEA) approved. Part one of the study looks at what, if anything, was discussed with respect to infertility with the cancer patient and what risk of infertility was given. Part two of the audit details information on the quality of material stored, how it was obtained and what was discussed. Results of this study should be available next year but pilot interviews with adolescent males by Glaser et al. [50] have revealed a number of common themes. Patients strongly believe their choice is of paramount importance and they strongly feel the need for information relating to sperm storage. Many patients sought increased input on the significance of fertility preservation and stressed the importance of communication with professionals on this subject. In addition to this, a common theme was that patients felt extremely pressurised about making decisions with respect to sperm storage in this setting.

### 7.2. The Legal Aspects of Fertility Preservation Young Cancer Patients

At present in the United Kingdom, a young person greater than 16 years of age is presumed capable of giving valid consent for treatment and removal of gametes (under common law) and storage and use of these gametes (governed by the HFE Act of 1990). A young person less than 16 years of age is presumed not capable of giving consent for treatment, removal of gametes and/or storage and use of these gametes. However, cases do exist where patients less than 16 years of age have been able to demonstrate capacity to undertake valid consent for this, in other words Gillick competent [51] is allowed to, in accordance with the 1990 [52]. Act as long as he is fully informed and understands the proposed line of treatment, benefits and attendant risk. A further issue is that young people less than 16 years of age who are not deemed competent can rely on their parents to give consent for medical procedures that are deemed to be in their children's best interest. Unfortunately, parents are not allowed to give consent for the storage and use of gametes, and therefore there is currently no option to preserve fertility in the prepubertal boy.

Experimentation is underway looking at the storage and use of gonadal tissue from children [47], however this has major ethical issues, not least of which is consent (which is only valid if it is voluntarily obtained from an informed, competent person). Proxy consent can be undertaken in a therapeutic setting if it is deemed to be in the best interests of the patient, but the question remains as to whether removal of gonadal tissue is actually fulfilling this criterion. The Human Fertilisation and Embryology Authority (HFEA) 1990 Act has jurisdiction over the storage and use of live human gametes and embryos created in vitro, and by definition a gamete is a “reproductive cell with a haploid set of chromosomes that is able to take part in fertilisation with another of the opposite sex to form a zygote”. The implications of this Act mean that no licence is required to store gonadal tissue from prepubertal children because they do not contain gametes and therefore primordial follicles in the cortical strips of ovaries (which are not considered gametes) may be stored for girls whose parents consent on their child's behalf because they believe that retrieval and storage of this tissue is in the girl's best interests. Unfortunately the same does not apply to boys, and although boys with Tanner Stage 2 [53] or greater development may have tissue stored in accordance with the 1990 Act if they can give written informed consent, parents are not allowed on their child's behalf in this setting.

## 8. Fertility Treatment and Cancer Prognosis

A concern regarding assisted reproduction techniques is that women have to undergo hormonal manipulation, a possible association between the use of fertility drugs and the risk of specific cancers, this has not been convincingly demonstrated in epidemiologic studies. With regard to cancer risk in relation to the cause of subfertility, the only consistent association observed is an increased risk of endometrial cancer for women with subfertility due to hormonal disorders. While positive findings in some studies on fertility drugs and ovarian cancer risk have aroused serious concern, the associations observed in most of these reports appear to be due to bias or chance rather than being causal [54]. 

The link between ovarian cancer and fertility drugs has not been established and fertility therapy does not increase the risk of ovarian cancer in infertile patients who already have an increased baseline risk as a result of their infertility [55]. 

However it was reported at the Society of Gynecologic Oncologists Annual Meeting that, the long-term risk for invasive ovarian cancer among women receiving treatment for IVF compared with subfertile women not treated (relative risk 1.51) and for borderline tumors, was increased (relative risk 4.40). Ovarian stimulation for in vitro fertilization (IVF) may increase the risk of ovarian malignancies, especially borderline tumors of the ovary, according to findings from a 15-year followup of a large Dutch cohort study [56].

## 9. Pregnancy and Its Effect on Different Cancers

The studies support a protective relationship between parity and the incidence of ovarian cancer [57–59]. Nulliparity increases the baseline risk twofold. Infertility appears to increase the risk in those patients who do not subsequently conceive [60–62]. 

 Also in breast cancer nulliparity is associated with an increased risk and parity reduces the risk, although a Swedish [63] and Norwegian [64] study provided an evidence that the risk of breast cancer is transiently increased after pregnancy followed by subsequent decrease in risk; also early menarche and late age at first pregnancy are associated with increased risk [65]. Parity (High progesterone dose in pregnancy) is a protective whereas nulliparity and anovulatory cycles are the risk factor for endometrial cancer.

## 10. Summary

Improved cancer care associated with increased cure rates and long-term survival, coupled with advances in fertility treatment means that it is now imperative that fertility preservation is considered as part of the care offered to these patients. This can only be approached within a multidisciplinary setting. There are obvious challenges that still remain to be resolved, especially in the area of fertility preservation in prepubertal patients. These include ethical issues, such as valid consent and research in the area of tissue retrieval, cryopreservation and transplantation.

## Figures and Tables

**Table 1:** 

Agent	Known effect on testis
Cyclophosphamide	Severe
Nitrogen mustard	Severe
Procarbazine	Severe
Bleomycin	Moderate
Carboplatin	Moderate
Cisplatin	Moderate
Cytarabine	Moderate
Doxorubicin	Moderate
Etoposide	Moderate
Ifosfamide	Moderate
Thioguanine	Moderate
Vinblastine	Moderate
Vincristine	Moderate
Methotrexate	Minimal

Effects are dose dependent.

Severe = Azoospermia shortly after treatement with less than 20% recovery of spermatogenesis.

Moderate = possible azoospermia shortly after treatment with 20–50% of patients recovering spermatogenesis.

Minimal = possibility of transient azoospermia but more than 50% of patients recovering spermatogenesis.
